# The CARD9 Gene in Koalas (*Phascolarctos cinereus*): Does It Play a Role in the *Cryptococcus*–Koala Interaction?

**DOI:** 10.3390/jof10060409

**Published:** 2024-06-06

**Authors:** Hannah P. Newton, Damien P. Higgins, Andrea Casteriano, Belinda R. Wright, Mark B. Krockenberger, Luisa H. M. Miranda

**Affiliations:** Sydney School of Veterinary Science, Faculty of Science, The University of Sydney, Sydney, NSW 2006, Australia; hannah.newton@sydney.edu.au (H.P.N.); damien.higgins@sydney.edu.au (D.P.H.); andrea.casteriano@sydney.edu.au (A.C.); belinda.wright@sydney.edu.au (B.R.W.); mark.krockenberger@sydney.edu.au (M.B.K.)

**Keywords:** *Cryptococcus*, *C. gattii*, Caspase recruitment domain-containing protein 9 (CARD9), koala (*Phascolarctos cinereus*), fugal immunity, innate immunity

## Abstract

*Cryptococcus* is a genus of fungal pathogens that can infect and cause disease in a range of host species and is particularly prominent in koalas (*Phascolarctos cinerus*). Like other host species, koalas display a range of outcomes upon exposure to environmental *Cryptococcus,* from external nasal colonization to asymptomatic invasive infection and, in rare cases, severe clinical disease resulting in death. Host factors contributing to these varied outcomes are poorly understood. Due to their close relationship with eucalypt trees (a key environmental niche for *Cryptococcus gattii*) and suspected continual exposure to the pathogen, koalas provide a unique opportunity to examine host susceptibility in natural infections. Caspase recruitment domain-containing protein 9 (CARD9) is a key intracellular signaling protein in the fungal innate immune response. Humans with mutations in CARD9 succumb to several different severe and chronic fungal infections. This study is the first to sequence and explore CARD9 variation in multiple koalas using Sanger sequencing. Four CARD9 exons were successfully sequenced in 22 koalas from a New South Wales, Australia population. We found minimal variation between koalas across all four exons, an observation that was also made when CARD9 sequences were compared between koalas and six other species, including humans and mice. Ten single-nucleotide polymorphisms (SNP) were identified in this study and explored in the context of cryptococcal exposure outcomes. While we did not find any significant association with variation in cryptococcal outcomes, we found a high degree of conservation between species at several SNP loci that requires further investigation. The findings from this study lay the groundwork for further investigations of CARD9 and *Cryptococcus* both in koalas and other species, and highlight several considerations for future studies.

## 1. Introduction

Cryptococcosis is an important systemic mycosis affecting a wide range of host species and is predominantly caused by *Cryptococcus neoformans* and *Cryptococcus gattii* species complexes [1]. Koalas (*Phascolarctos cinereus*) have one of the highest prevalence of clinical cryptococcal infections of any host species, especially in captivity [2,3,4]. This is likely the result of the strong association between *C. gattii* spp. complex (specifically VG1/*C. gattii* sensu stricto) and eucalypt trees, which provide a distinct interface between this pathogen and koalas. This has led to *C. gattii* spp. complex becoming the dominant colonizing and infective species in koalas [4,5]. The high prevalence of disease in koalas has been attributed to this association, and it is suspected that individuals are continuously exposed to infectious elements throughout their lifetime [6,7,8,9,10]. While environmental burden is a strong contributor to disease in captive koalas [3,11], it may not completely explain patterns of mycosis in wild koala populations [10].

Diseases caused by *Cryptococcus* spp. are generally initiated through inhalation of infectious propagules from the environment, followed by colonization of the upper respiratory tract and, in some cases, the progression to locally and/or systemically invasive subclinical or clinical disease [11,12,13]. Progression to clinical disease is not linear, with all potential host species demonstrating variable and dynamic changes in the host–pathogen interaction within individuals [2,6,14,15]. Colonization of the upper respiratory tract is the first part of the interaction between the fungus and the host [4,5], which may proceed to limited invasion without clinical signs [6]. Invasion of the mucosal barrier and primary line of immune defenses in the host is evident by the detection of cryptococcal antigens in the bloodstream [6,9,11]. This may progress in some koalas to more extensive tissue invasion accompanied by clinical signs [7]. As is common to all other hosts, clinical disease in koalas is the least observed of these outcomes. If left untreated, cryptococcosis is fatal; however, these variations in early host–pathogen interactions are still poorly understood [1,16]. The koala is a relatively sedentary animal with a close relationship to an environment in which *C. gattii* is often present, providing a suitable natural model for investigation of the host–pathogen–environment interaction.

Successful host response to *Cryptococcus* species complexes is greatly dependent on the overall immune status of the host and the correct activation of antifungal innate and adaptive immune cells. The innate immune response to initial exposure and appropriate coordination of a fungi-specific adaptive immune response is critical for the elimination and suppression of the invading cryptococcal cells [17,18]. Successful activation of such a response begins with the recognition of cryptococcal cells by fungal-specific pattern recognition receptors (PRRs) on the surface of macrophages, stimulating a complex but highly targeted intracellular signaling cascade. This signaling cascade results in the direction of classical macrophage activation (CMA), which subsequently activates T-helper 1 (Th-1) and Th-17 cells in the adaptive response, leading to pathogen elimination and reduced tissue invasion [16,18,19]. Impaired T-cell functioning and general immunosuppression are key risk factors for cryptococcosis. However, disease caused by *C. gattii* spp. complex typically occurs in apparently immunocompetent hosts, an association that is still not fully understood [20,21]. As the *C. gattii* spp. complex is the predominant disease-causing species in koalas, and the innate immune system plays a key role in directing cryptococcal immunity; investigating components of the innate immune response as possible causes of susceptibility to this species provides an attractive avenue of inquiry. Uncovering potential genetic susceptibilities to cryptococcal infections in hosts like the koalas may allow for more comprehensive management options in susceptible individuals and alter breeding practices or translocation screening of captive koalas where cryptococcosis is more common. Identifying genetic contributors will also provide some insight into *C. gattii* pathogenesis in apparently immunocompetent hosts. 

Caspase recruitment domain-containing protein family member 9 (CARD9) is a key intracellular signaling protein with a fundamental role in connecting innate and adaptive immune responses against fungal pathogens [22,23,24]. Several fungal-specific PRR intracellular signaling cascades converge on the activation of CARD9 [22]. The main functional component of CARD9 is made up of a caspase-binding domain (CBD), a linker region and the N-terminal end of a coiled-coil domain (CCD) [18,24,25]. Once activated, this region binds to two other proteins to propagate the signal, culminating in the release of cytokines and chemokines that direct CMA, Th-17 and, to a lesser degree, Th-1 activation [22,24,25].

There are currently 29 deleterious CARD9 mutations described in human patients with chronic and severe mycoses [26,27,28]. The effects of these mutations appear to be extremely fungal-specific. Experimental mouse models have demonstrated the importance of a functioning CARD9 protein for cryptococcal elimination and avoidance of disease [18,24]. While this gene is yet to be validated in koalas, the phenotype of CARD9 mutations in humans and mice, together with its role in fungal immunity, highlight it as an ideal candidate for exploration of host-related susceptibility to cryptococcosis in the koala. 

Here, we explore the potential for CARD9 mutations to cause differences in exposure outcomes to *Cryptococcus* spp. in a free-ranging koala population from New South Wales (NSW). Diversity in the genetic sequence of four CARD9 exons was explored in this koala population in the context of cryptococcal exposure outcomes (negative, colonization, and invasive disease). These exons code for the CBD and N-terminal CCD and were selected based on previous reports of CARD9 mutations in humans. This line of inquiry is not only relevant for the genetic management and risk assessment for cryptococcosis in koalas but may have extended implications in areas such as Canada, where *C. gattii* infections are on the increase and are spreading into novel animal and human populations [29]. As CARD9 has been studied only rarely in animals other than humans and model organisms, we also interrogated the diversity of these CARD9 exons among a selection of other species to better contextualize any variation observed in the koala gene. This study provides a preliminary investigation into CARD9 mutations as a causative gene in host susceptibility to natural cryptococcal exposure in koalas and provides a key foundation for further study in this area.

## 2. Materials and Methods

### 2.1. Study Population and Samples

DNA extracted previously (DNeasy Blood & Tissue Kit, QIAGEN, Hilden, Germany) from whole blood samples of free-ranging koalas in the Gunnedah Shire in Liverpool Plains, New South Wales (NSW), Australia (30°59′ S, 150°16′ E) and stored at −20 °C, were used in this study. This koala population is periodically screened for *Cryptococcus* infection by assessing nasal colonization and antigenemia. Nasal colonization was verified through fungal culture from nasal swabs; species assessment of positive cultures through Matrix-assisted laser desorption/ionization-time of flight (MALDI-TOF) mass spectrometry (MS); and detection of *Cryptococcus* antigenemia was performed by Lateral Flow Assay (LFA) in serum samples. Positive LFA tests were confirmed by a latex cryptococcal antigen agglutination test (LCAT), and an endpoint titer was determined. These results were then used to classify koalas into study groups: colonized, subclinical cryptococcosis, and negative. Koalas were classified as colonized based on positive fungal culture in at least one sampling time point and negative LFA tests on all sampling time points. Koalas were considered subclinical if they had a positive LFA test on at least one collection time point, regardless of fungal culture results. Koalas were considered negative for *Cryptococcus* if they were negative for *Cryptococcus* screening (negative fungal culture and LFA) at all time points. Koalas with negative results for both colonization and LFA at all sampling time points were classified as negative. All koalas with nasal colonization (sampled between 2020 and 2022) and with antigenemia (sampled between 2016 and 2022) were included in this study. Additional negative cases were randomly selected from the koalas screened between 2020 and 2022 to comprise a negative group. All procedures involving sample collection were approved by the University of Sydney AEC under the approval number 2019/1547. 

Cases of clinical cryptococcosis were selected from the archive of the Veterinary Pathology Diagnostic Services, Sydney School of Veterinary Science, Faculty of Science, The University of Sydney, based on the detection of intralesional cryptococcal yeasts by histopathology. These samples were formalin-fixed, paraffin-embedded (FFPE) archival tissues previously submitted for diagnosis. DNA was extracted from these FFPE tissues using the QIAamp^®^ DNA Mini Kit (QIAGEN, Hilden, Germany) as per manufacturer instructions. These cases were considered alongside those from the subclinical cryptococcosis group and analyzed as a combined “invasive” group.

The final number of koalas used for our investigation of *Cryptococcus* exposure outcomes was 22 koalas.

### 2.2. CARD9 Primer Design 

The koala genomic sequence predicted to represent the CARD9 gene was extracted from NCBI (GCF_002099425.1, Gene ID:110205341) and used for primer design. QIAGEN CLC Main Workbench version 22 software was used for all sequence analyses. All alignments were done using the “Alignment tool” [Gap open cost = 10; gap extension cost = 1.0; end gap cost = cheap; alignment = very accurate], and percentage similarities were calculated using the “Create Pairwise Comparison” tool. Genomic coding regions used for primer design are described in Table 1.

The koala CARD9 gene is 35,417 base pairs long and consists of 14 exons. Due to its large size, exons 2, 3, 5, and 6 of the koala CARD9 gene were selected for analysis based on the human CARD9 gene, as these four exons code for the CBD and N-terminal region of the CCD functional domains of the CARD9 protein. This region of the protein also contains several key amino acids involved in protein stabilization and functioning [25,30]. In addition, these four exons contain 22 of the 29 currently described deleterious CARD9 mutations with causal links to susceptibility to fungal diseases in humans [28].

Forward and reverse primers for each exon were designed using the NCBI Primer Design tool (https://www.ncbi.nlm.nih.gov/tools/primer-blast/, accessed on 13 May 2021) and visual inspection of the target sequences, with a total of 16 primers designed (Table A1). The specificity of each primer for the target sequence was assessed by a nucleotide BLAST search against the koala genome. Primers were then sourced from MACROGEN, Inc. (Seoul, Republic of Korea). The final primer pairs used for this study were chosen based on the cleanest electrophoresis band and can be found in Table 2.

### 2.3. PCR Protocol

Primers were optimized using a sample of archived koala DNA (University of Sydney, NSW, Australia) as a template. The reaction volume was 25 µL with final concentrations of 1× Q5 high-fidelity DNA polymerase, 200 µM each dNTP, 2.0 mM Mg++ (2× Q5^®^ High Fidelity Master Mix, New England BioLabs, Notting Hill, Australia), 0.5 µM of each primer and DNA template (5 µL of unknown concentration). Optimal thermocycler conditions consisted of an initial denaturation at 98 °C for 30 s followed by 35 cycles of denaturation at 98 °C for 10 s, annealing at 63 °C for 20 s, and extension at 72 °C for 10 s, with a final extension at 72 °C for 2 min. For DNA extracted from FFPE samples, PCR reactions were extended to 40 cycles following the same protocol described, as recommended by the DNA extraction-kit manufacturer (QIAamp^®^ DNA mini kit, QIAGEN, Hilden, Germany). Successful amplification was determined by electrophoresis on a 2% (*w*/*v*) TBE agarose gel. 

### 2.4. Sanger Sequence Analysis

PCR products were sent for DNA purification and bidirectional Sanger sequencing at MACROGEN (Seoul, Republic of Korea) using the same PCR amplification primers. Analysis of Sanger sequences was performed using CLC Main Workbench v.22 (QIAGEN, Hilden, Germany) software. Single-nucleotide polymorphisms (SNPs) were identified as either heterozygous peaks or a single homozygous alternative peak on the chromatogram. Heterozygous peaks were counted if the second peak was 50–100% the size of the first. Regions of poor sequence quality were excluded. 

The CLC Main Workbench v.22, ‘Assemble Sequences to a Reference’ tool [Minimum aligned read length = 50, alignment stringency = medium, conflict resolution = vote (A, C, G, T)] aligned forward and reverse strands of each sample with the same reference sequence used in the primer design process. The consensus sequences of each sample were then aligned with the reference sequence and compared. Points of variation were identified, and the genotype of each koala sample was noted. 

### 2.5. CARD9 Sequence Comparison between Species 

To assess how variation within the koala CARD9 gene compares with variation between CARD9 sequences of different species, the predicted koala CARD9 sequence was aligned with predicted CARD9 sequences from six other species obtained from the NCBI online database. Human (*Homo sapiens*; Gene ID: 64170) and mouse (*Mus musculus*; GCF_000001635.27, Gene ID: 332579) CARD9 sequences were selected for comparison as they have been widely described and verified. Sequences from the following four marsupial species were also used: common brushtail possum (*Trichosurus vulpecula*; GCF_011100635.1, Gene ID: 118843096), Tasmanian devil (*Sarcophilus harrisii*; GCF_902635505.1, Gene ID: 100931004), common wombat (*Vombatus ursinus*; GCF_900497805.2, Gene ID: 114048523), and grey short-tailed opossum (*Monodelphis domestica*; GCF_000002295.2, Gene ID: 100020522). Sequence identities for these marsupial species are predicted and are yet to be verified. QIAGEN CLC Main Workbench version 22 software was used for all sequence analyses in the same manner described for the CARD9 primer design. Variation was observed in the number of exons in the CARD9 gene of the different species examined. To confirm comparisons were made between exons most similar to each other, exons number 2, 3, 5, and 6 from the koala genomic sequence were aligned with the CARD9 sequence of each species investigated and the exon that shared the most sequence similarity with the target koala exons were used for exon comparison between species. The exons used in this analysis are outlined in Table 3. Results of comparisons are described in relation to koala exons 2, 3, 5, and 6.

Variation of the CARD9 protein sequence was also assessed using residues 1 to 317, as coded by exons 2 to 6 of the koala CARD9 gene. This region of amino acid sequence was isolated and aligned for each of the seven species (koala, XP_020837556.1; human, NP_434700.2; mouse, XP_006498198.1; common brushtail possum, XP_036606396.1; Tasmanian devil, XP_003757538.1; common wombat, XP_027725300.1; grey short-tailed opossum, XP_007476965.1). Residues affected by deleterious amino acid mutations previously described in humans and residues involved in protein function were then highlighted on this alignment [24,27,28,30].

### 2.6. Analysis

Koalas were included for analysis if the SNP locus was covered by both the forward and reverse Sanger sequences. For statistical analysis, each SNP found during Sanger sequence analysis was considered either present or absent in samples. Fisher’s exact test was used to compare individual CARD9 genotypes (presence or absence of individual SNPs) among the *Cryptococcus* infection status (negative, nasal colonization, or invasive).

## 3. Results

### 3.1. Cryptococcus Exposure Outcomes

Based on the presence of *Cryptococcus* nasal colonization and/or positive LFA in samples collected over up to five different time points, 13 koalas in the Gunnedah Shire region of NSW were included in the study: 7 out of 76 koalas examined between 2020 and 2022 were classified as colonized, and 6 out of 266 koalas examined between 2016 and 2022 were included in the subclinical group. Five of these subclinical koalas had concurrent nasal colonization. Two clinical cases of fatal cryptococcosis in wild koalas attended at Port Stephens Hospital and whose necropsy specimens were stored in the VPDS archive, were included and, together with the six subclinical cryptococcosis cases, formed the invasive infection group (*n* = 8). Seven negative controls were randomly selected from the 63 koalas that tested negative for *Cryptococcus* screening (negative fungal culture and LFA) at all time points between 2020 and 2022. 

Five of the thirteen colonized and subclinical cryptococcosis koalas returned different test results at different time points, either progressing from negative to positive or regressing from positive to negative (Table 4). Only one koala (USYD075F) had re-occurring nasal colonization: it was sampled at all five time points, with nasal colonization detected at sampling time points one and five. 

The colonizing species were identified for most koalas presenting nasal colonization (*n* = 10) (Table 4), consisting of the seven koalas in the colonized group and three koalas from the subclinical group. *C. neoformans* was the dominant colonizing species in the nasal cavity of the colonized group (3/7). Only three subclinical koalas with concurrent nasal colonization had the species identified. All three were colonized by *C. gattii*. 

### 3.2. CARD9 Sequencing Results and SNP Identification 

Out of the 22 koalas included in this study, the CARD9 target exons were amplified from all but two, but some were excluded from the final analysis due to poor sequence quality. A total of 19, 18, 21, and 22 koalas were included for final sequence analysis of exons 2, 3, 5, and 6, respectively (which koalas were sequenced and included for each exon are described in Table 4). Primers used for exons 2 and 3 did not amplify the two koala samples extracted from FFPE tissues (21-1137 and 14-1684). Dilution of inhibitors and increasing extension time from 10 to 20 s at 72 °C were attempted but did not improve amplification in affected FFPE samples. 

A total of one SNP and nine putative SNPs were found in exon 2 and exon 6, respectively, and no sequence variation was observed in exons 3 and 5. The alternate allele was present in more than one-quarter of the koalas analyzed for eight out of ten of the identified SNPs (Table 5). SNPs NW_018343963.1:19,355,780G > A(p.S23S), NW_018343963.1:19,369,197C > G(p.K317N) and NW_018343963.1:19,369,212C > A(p.E312N) were seen in more than half of the sampled koalas (Table 5). The SNP in exon 2 and five of the putative SNPs in exon 6 are in exon coding regions. The SNP identified in exon 2, NW_018343963.1:19,355,780G > A(p.S23S), was observed in heterozygous and homozygous koalas but was found to be a synonymous mutation at p.S23 and was excluded from statistical analysis of disease outcomes.

**Figure 1:**
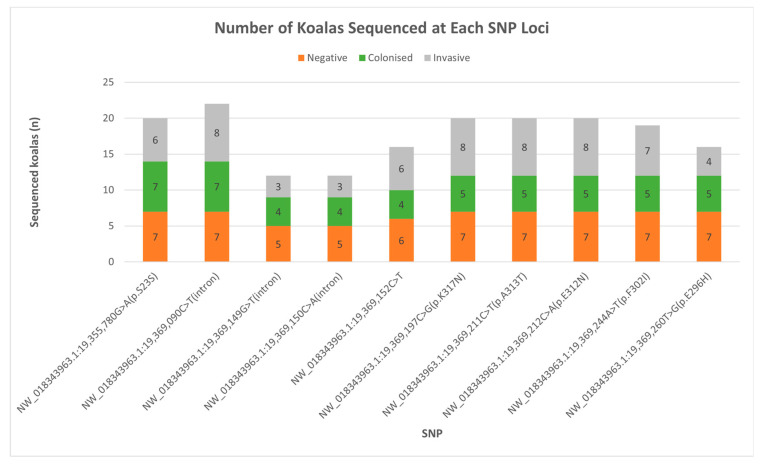
Number of koalas analyzed at each SNP/putative SNP locus according to study grouping. Grey: Invasive group. Green: Colonized group. Orange: Negative group.

**Figure 2:**
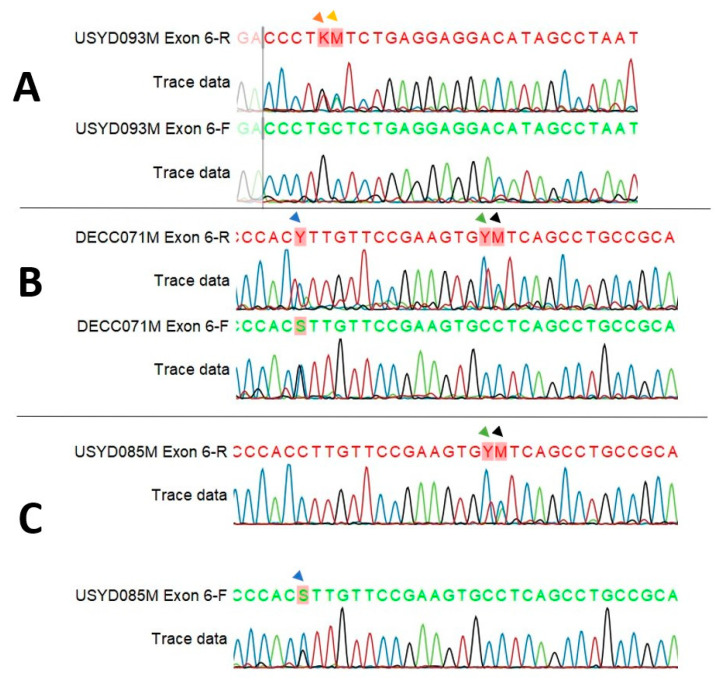
Excerpts from Sanger sequencing chromatograms highlight five of the nine putative SNPs identified in exon 6. Heterozygous peaks are highlighted by arrows in koalas: USYD093M (**A**), DECC071M (**B**), and USYD085M (**C**). Koala DECC071M (**B**) has heterozygous peaks at SNP NW_018343963.1:19,369,197C > G/T in both forward and reverse sequencing strands. Orange arrow: NW_018343963.1:19,369,149G > T(intron). Yellow arrow: NW_018343963.1:19,369,150G > A. Blue arrow: NW_018343963.1:19,369,197C > G(p.K317N). Green arrow: NW_018343963.1:19,369,211C > T(p.A313T). Black arrow: NW_018343963.1:19,369,212C > A(p.E312N). Exon 6-R: Reverse sequence. Exon 6-F: Forward sequence.

After sequence trimming, the coding region for amino acids p.290–317 in exon 6 was reliably sequenced. Five of the nine putative SNPs in this exon occurred in this coding region, and all five cause missense mutations, the remaining four occur in the flanking intronic region. The putative SNPs at either end of the exon 6 amplicon had fewer koalas used in the final analysis due to poor sequence quality at either end of the amplicon in some koalas (Figure 1). Eight of the nine putative SNPs were only observed as heterozygous peaks in one sequencing strand, despite clean sequence coverage at each locus in both forward and reverse stands (Figure 2). Discordant sequencing results were observed when an alternative primer was used, see Appendix A. SNP NW_018343963.1:19,369,090C > T(intron) was significantly impacted by the lack of coverage in one sequencing strand. The forward sequence in all samples did not cover this locus. Due to the absence of a forward strand for comparison in all samples and provided the sequence was clean at and around NW_018343963.1:19,369,090C > T(intron), the sample was included for preliminary analysis. Koala USYD038F from the negative group had a well-defined, single peak for the alternate allele at this locus and several other koalas had clear heterozygous peaks. 

### 3.3. Investigating Described SNPs in Relation to Cryptococcus Exposure Outcomes

**Figure 3:**
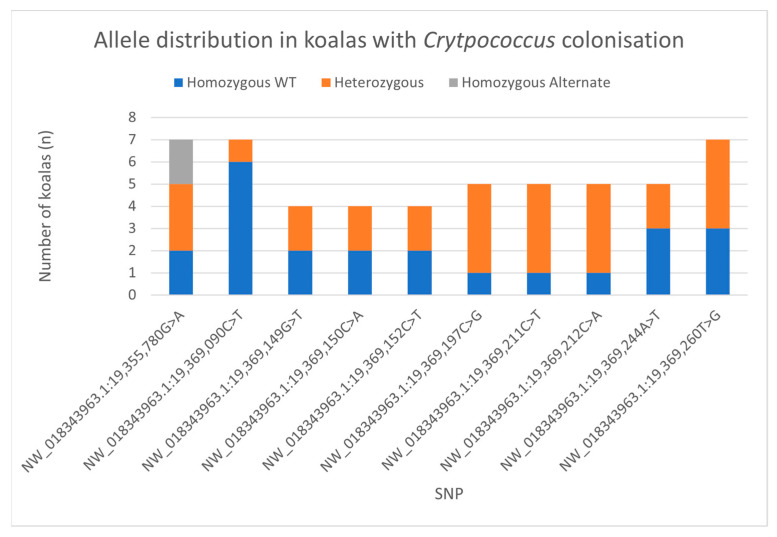
Distribution of SNP alleles in colonized koala group. Blue: Homozygous wild-type allele (WT). Orange: Heterozygous allele. Grey: Homozygous alternate allele. NW_018343963.1:19,355,780G > A(p.S23S) (*n* = 7), NW_018343963.1:19,369,090C > T(intron) (*n* = 7), NW_018343963.1:19,369,149G > T(intron) (*n* = 4), NW_018343963.1:19,369,150C > A(intron) (*n* = 4), NW_018343963.1:19,369,152C > T(intron) (*n* = 4), NW_018343963.1:19,369,197C > G(p.K317N) (*n* = 5), NW_018343963.1:19,369,211C > T(p.A313T) (*n* = 5), NW_018343963.1:19,369,212C > A(p.E312N) (*n* = 5), NW_018343963.1:19,369,244A > T(p.F302I) (*n* = 5), NW_018343963.1:19,369,260T > G(p.E296H) (*n* = 7).

The heterozygous alleles for seven of the nine putative SNPs were observed in more than half of the colonized group analyzed at each locus (Figure 3). This was high compared with only one and four putative SNPs occurring in more than half of the negative and invasive groups, respectively. The colonized koalas were also the only study group with all ten SNPs. SNP NW_018343963.1:19,369,152C > T(intron) was only seen in two colonized koalas, DECC015M and USYD006M. These two koalas were also the two with the most putative SNPs present in exon 6, 9, and 8, respectively. 

Some SNPs were not present in some koala groupings. NW_018343963.1:19,369,152C > T(intron) was not present in the invasive group. SNP NW_018343963.1:19,369,260T > G(p.E296H) and homozygous alternative NW_018343963.1:19,355,780G > A(p.S23S) koalas were absent from the negative group. When looked at in isolation, the two clinically infected koalas did not have any of the SNPs observed in exon 6. The absence of these SNPs and putative SNPs in certain groups was not found to be statistically relevant. 

### 3.4. CARD9 Sequence Comparison between Species

**Figure 4:**
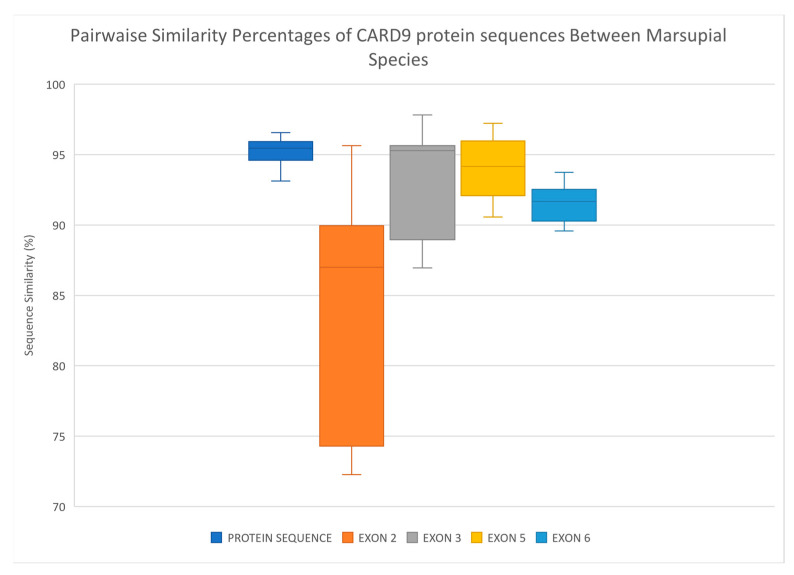
Box plot illustrating the spread of sequence similarity percentages for the five marsupial species examined through pairwise comparison of CARD9 protein sequences (region p1–320; Dark Blue), and CARD9 genomic sequences for exon 2 (Orange), exon 3 (Grey), exon 5 (Yellow), and exon 6 (Light Blue). Species compared: koala (*Phascolarctos cinereus*), common brushtail possum (*Trichosurus vulpecula*), grey short-tailed opossum (*Monodelphis domestica*), Tasmanian devil (*Sarcophilus harrisii*), and common wombat (*Vombatus ursinus*).

Due to the large differences in length of the CARD9 genomic sequence and the high intronic burden of this gene, a whole gene comparison was not possible between species. Instead, the predicted genomic sequences for the target exons (exons 2, 3, 5, and 6) in the koala CARD9 gene showed a 57–78% similarity with corresponding exons in the verified human and mouse genes. The target exons were more similar among predicted CARD9 genomic sequences of four other marsupial species based on pairwise comparison, with similarity percentages ranging 72–98% (Figure 4). As shown in Figure 4, exon 2 was the most variable among marsupial species, while exons 3 and 5 had the highest average similarities among marsupial species. 

**Figure 5:**
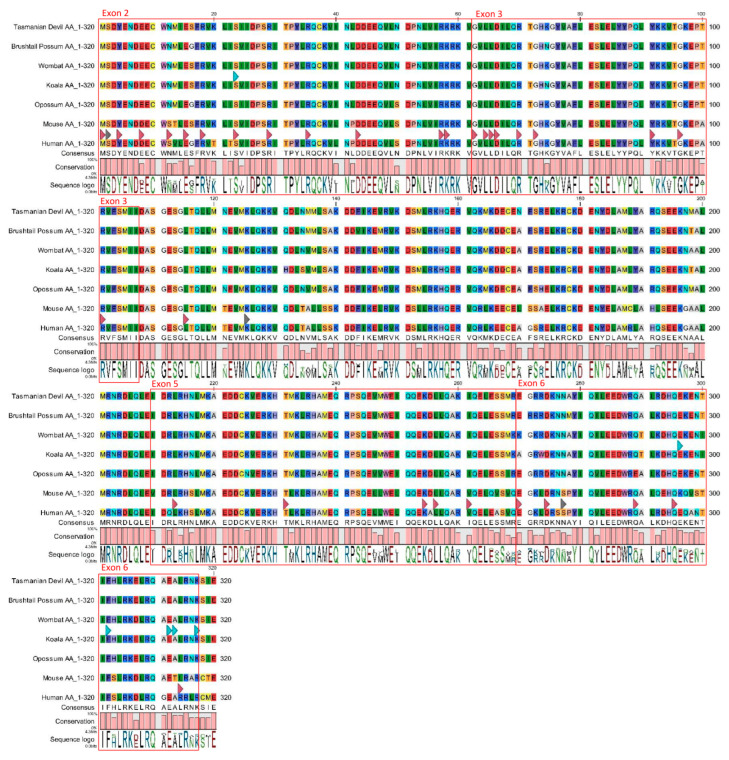
Amino acid alignment of the CARD9 gene (amino acids (aa) 1–360) for seven different species; koala (*Phascolarctos cinereus*), common brushtail possum (*Trichosurus vulpecula*), human (*Homo sapiens*), mouse (*Mus musculus*), grey short-tailed opossum (*Monodelphis domestica*), Tasmanian devil (*Sarcophilus harrisii*), and common wombat (*Vombatus ursinus*). Regions coded for by exons 2, 3, 5, and 6 are outlined in red boxes and labeled accordingly. Red arrows = aa affected by disease-associated mutations in humans [27,28]. Grey arrows = aa that are phosphorylated or ubiquitinated [25]. Blue arrows = aa affected by six single-nucleotide polymorphisms (SNPs) identified in this study. The SNP and five putative SNPs found in this study and highlighted in this image are (in sequential order, left to right); NW_018343963.1:19,355,780G > A(p. S23S); NW_018343963.1:19,369,260T > G(p.E296H); NW_018343963.1:19,369,244A > T(p.F302I); NW_018343963.1:19,369,212C > A(p.E312N); NW_018343963.1:19,369,211C > T(p.A313T); NW_018343963.1:19,369,197C > G(p.K317N).

Pairwise comparison of predicted amino acids 1 to 320 amongst the seven species (koala, human, mouse, common wombat, Tasmanian devil, opossum, and brushtail possum) (Figure 5) showed percentage similarities ranging 80–97%. Sequence similarities were reflective of taxonomic relationships with human and mouse sequences, demonstrating low percentage similarities when compared with each of the five marsupial species (80–81%) and closely related species such as the koala and wombat showing a high degree of similarity (97%). Overall, the protein sequences between species were more similar, and similarity scores were less variable (range 3.44%) than the genomic sequences for each corresponding exon (Figure 4). 

Of the 35 amino acids with described mutations in humans [27,28] or roles in CARD9 functioning [24,30], 80% (28/35) were conserved across the predicted sequences for all seven species when the CARD9 protein sequences were compared (Figure 5). The six SNPs in exon coding regions were also compared among CARD9 protein sequences of different species (Figure 5). Two out of five of the putative SNPs in exon 6 affect amino acids that are otherwise conserved across the CARD9 protein sequences of all seven species examined, and the remaining three were conserved across all marsupial species.

## 4. Discussion

This study is the first to successfully amplify and sequence key regions of the CARD9 gene in multiple koalas, leading to the detection of ten SNPs—one confirmed SNP and nine putative SNPs found across two exons. Except for these ten SNPs, minimal variation was found between sampled koalas and the reference sequence used in all four CARD9 exons. Sequence conservation was also observed when CARD9 exon sequences were compared among several species, suggesting that variation is poorly tolerated in this region of the gene and supporting this gene as a key intracellular signaling protein [26]. All putative SNPs were reproducible within the same koala and across several different animals, providing evidence of the validity of these putative SNPs and a basis for further exploration of these loci in koalas. Missense mutations among several putative SNPs, as well as the absence of homozygous mutants at putative SNP loci, suggest potential for disrupted functioning. Thus, while we found no association between CARD9 mutations and varied outcomes to *Cryptococcus* in this study, our results can neither support nor deny CARD9 involvement in the susceptibility of koalas to cryptococcosis or other fungal infections. We advocate for further investigations into the role of CARD9 in cryptococcal infection and highlight several other avenues for ongoing research. Our findings provide additional support for the continued presence of *Cryptococcus* in the koala, and further support previous work demonstrating *C. gattii* remains the dominant colonizing species.

As the first study to sequence CARD9 in multiple koalas, our results validate the predicted CARD9 sequence for koalas provided by the NCBI database. We observed minimal sequence variation between the koalas studied and the predicted sequence used as a reference in this study. A high degree of sequence similarity was also observed between species, even though the CARD9 gene for several other marsupial species was also labelled as predicted on the NCBI database. This observation demonstrates that these predicted sequences would provide a reliable reference for future work on the CARD9 gene, both in koalas and the several marsupial species investigated here. This high degree of CARD9 conservation between species and reduced variation within koalas is likely reflective of its role as a key intracellular protein. Variation is likely to be poorly tolerated in this gene, as the functioning of CARD9 and its role in connecting the innate and adaptive immune response to fungal pathogens is dependent on highly targeted, intracellular protein-protein interactions [18,23,25]. Our observations here in koalas support the assumption that these four exons play a key role in the overall functioning of CARD9 protein and is further strengthened by the 76% (22/29) of deleterious mutation in human patients with fungal disease occurring in these exons [28]. These findings also build on and support previous work by Glocker et al. (2009) [26], showing CARD9 is conserved across humans and mice. In addition, CARD9 protein sequences were more conserved across these species than when comparing individual genomic coding exons. This would suggest that much of the variation observed in the genomic exon sequences of different marsupial species are likely synonymous substitutions. Conservation of key CARD9 exons across species may be used as a basis to explore this gene as a source of susceptibility to fungal pathogens in other species and may act as further evidence that missense mutations in these four exons are poorly tolerated.

Despite the discordance between sequencing strands, seven of the nine putative SNPs identified here were observed in more than one-quarter of koalas sequenced (two SNPs in more than half), and SNPs were reproducible both within the same koala and across several different PCR and sequencing reactions, thus excluding random PCR and sequencing errors as a potential cause, leading to the conclusion that they are true representations of variation. We hypothesize that the absence of putative SNPs in the complementary sequencing strand was due to allelic dropout (ADO). ADO is a well-described source of error in PCR-based sequencing methods and can result in inconsistencies in SNP detection, as seen in exon 6 [31,32,33]. ADO occurs when primers are unable to bind effectively to DNA when a particular SNP is present, preventing the amplification, sequencing, and detection of that allele. There are two main ways ADO can occur. First, if an SNP occurs in the binding region of the primer, the primer may not be able to bind as efficiently or at all in the presence of the SNP. Second, the shape of the DNA, which can be influenced by its sequence, would affect primer binding efficiency [34]. The complete absence of putative SNPs when samples from three koalas were re-sequenced with an alternative reverse primer for exon 6 (Appendix B) could be a further indication of ADO. While there is evidence for the legitimacy of these nine putative SNPs, to determine if ADO is the underlying cause of these results, new primers for exon 6 should be designed and tested. 

Of the five putative SNPs in exon 6 that cause missense mutations, we did not find any overlap with CARD9 mutations described in humans susceptible to fungal infection. When deleterious mutations in humans were mapped against our CARD9 species alignment, affected amino acids were also conserved across the seven species, as seen with the five SNPs identified here. The synonymous SNP in this study (NW_018343963.1:19,355,780G > A(p.S23S)) was found to overlap with one human mutation at p.S23. This serine residue is affected by a premature stop codon observed in a human patient, p.S23X [35]. This p.S23X mutation has only been described in a single human patient and was observed as a compound heterozygous mutation, something that was not observed with our synonymous SNP found in koalas. The SNP identified here was also a synonymous mutation and, therefore, much less likely to affect protein functioning than a premature stop codon. However, it is worth noting that no homozygous alternative genotypes at NW_018343963.1:19,355,780G > A(p.S23S) were found in the negative group, and while there is no association with cryptococcal interactions in this instance, this SNP could be associated with another SNP or SNPs in exons not sequenced in this study. The otherwise high degree of conservation at missense putative SNP loci identified here could indicate a possible deleterious effect despite our study showing no association with susceptibility to cryptococcal infection. 

The lack of homozygous mutants at the five putative missense mutations could also be viewed as evidence supporting a potential effect on CARD9 functioning, assuming deleterious mutations related to fungal infection susceptibility in koalas behave similarly to those seen in humans. In humans, the impaired fungal immunity due to CARD9 mutation is only seen in homozygous mutants [28,30] or compound heterozygotes [35,36]. Family members of affected patients who are heterozygous carriers display little to no susceptibility to fungal infections [37,38,39]. If the five missense mutations we identified behave similarly, this may have contributed to the lack of association with exposure outcomes. As there are strong similarities between the CARD9 protein sequences of humans and koalas, particularly at the loci of these SNPs, one could hypothesize that these deleterious mutations in koalas may exhibit similar behavior. It is also worth noting that a high percentage of heterozygous individuals at seven of the nine putative SNP loci were observed in the colonized koalas when compared with the other study groups. It is possible that the small sample size confounded the significance of the presence of these putative SNPs in colonized koalas. However, it is also possible that colonization is not a true representation of early-stage infection; the transient nature of cryptococcal pathogenesis makes it hard to discern whether colonization is a precursor for invasive infection or whether colonization is an indicator of a successful response if it never progresses to invasive infection. These distinctions remain unclear within the field. 

Recurring colonization seen in koala USYD075F and changes in test outcomes seen in several other koalas over time is a clear demonstration of the transient nature of infections by this fungal pathogen. It is a feature also observed in several other host species [2,9,14]. This observation also means that our groupings are only snapshots of their state of pathogenesis and that koalas in the negative grouping may have been positive for nasal colonization or antigenemia outside of these times. This may have acted as a confounder and something that future studies utilizing a longitudinal design must take into account. Further investigations into genetic influencers of susceptibility to different cryptococcal exposure outcomes, such as colonization and subclinical disease, should consider a longitudinal study similar to the approach used here to ensure fluctuations in the stage of pathogenesis are accurately captured. The mechanisms involved in this transience are still not fully understood and must be considered when investigating this pathogen. It is also likely that *C. gattii* may influence this variability, as it is the dominant colonizing species in koalas (as shown here and in other studies [5,9,10]) and is known for infecting immunocompetent hosts. Investigations into host susceptibility to clinical cryptococcosis in koalas will also need to account for the low rate of clinical cryptococcosis (particularly in wild koalas) and may benefit from directly recruiting clinically diseased animals from veterinary settings or archival cases. The presence of individuals with heterozygous genotypes in this study demonstrates that there are mechanisms within the CARD9 gene of koalas for which homozygous mutant individuals may arise, with potential for functional implications. Additional work is needed on a larger sample size with more clinically diseased koalas to determine whether the hypotheses previously mentioned are accurate and ascertain if homozygous mutant individuals exist within koala populations.

Our investigation into CARD9 variation in koalas and its involvement in susceptibility to cryptococcal infections has not been able to demonstrate an association exists, and while we have outlined several reasons why this might be the case, further investigation is needed and other genes should also be considered. Immune-related genes such as B-cell leukemia 10 (BCL10), signal transducer and activator of transcription 3 (STAT3), protein kinase C-δ (PKCδ), or fungal PPRs such as Dectin-1 [24,40,41,42], all have been shown to influence host susceptibility to different fungal diseases and are also worth pursuing. Another immune factor for consideration in koalas is anti-granulocyte-macrophage colony-stimulating factor (Anti-GM-CSF) autoantibodies, as there is a demonstrated influence on cryptococcal infections in immunocompetent human patients [21,43]. As such, further work in the area should also try to implement a more wholistic, multivariant approach when investigating genetic susceptibility to *Cryptococcus* in koalas to ensure the challenges around small sample size seen here and other potential confounders such as disease transience are accounted for as much as possible.

## 5. Conclusions

Further investigations into CARD9 involvement in cryptococcal susceptibility are warranted as several putative SNPs identified here cause missense mutations, providing circumstantial evidence for an impact on CARD9 functioning. Future work should consider exploring these four exons in larger koala populations, extending the analysis to the ten other CARD9 exons not sequenced in this study and recruiting larger cohorts of clinically affected koalas. Improving our understanding of host-driven susceptibility to infections by *Cryptococcus*, such as genetic defects in proteins involved in fungal immunity, will allow for appropriate management options in susceptible hosts and provide a more comprehensive view of cryptococcal susceptibility. This study explored one potential gene for host susceptibility, and as described above, there are several other avenues yet to be explored. Recurring exposure, fluctuations in infection status over their lifetime and *C. gattii* as their primary infector make free-ranging koalas a perfect study model for natural infections by pathogenic *Cryptococcus* spp. complexes, provided issues of sample size can be addressed by investment in larger prospective studies.

The results of this study support such investment. Due to the broad range of host species, investigating disease influencers in wild koalas could provide insights into susceptibility in other hosts like humans, dogs, and cats with applications in both human and veterinary medicine. There is still much to learn about susceptibility to cryptococcosis, particularly in relation to infections by *C. gattii*, and ongoing research into koala-*Cryptococcus* interactions will prove to be a valuable avenue for future pursuits. Overall, the influences of host susceptibility to infections by *Cryptococcus* (more specifically. *C. gattii*) are still poorly understood, and we highlight the need for ongoing research in the field and the benefit of utilizing koalas as a model organism. Understanding how host susceptibility in koalas fits into the host–pathogen–environment interactions surrounding cryptococcosis will allow for not only a better understanding of the disease but also better genetic management of both wild and captive populations.

## Figures and Tables

**Table 1:** Sequences used for the design of primers targeting koala CARD9 exons 2, 3, 5, and 6.

Target Exon	Target Sequence	Coding Sequence	Length of Exon (nt)
Exon 2	19,375,488–19,376,222	19,375,665–19,375,922	258
Exon 3	19,374,974–19,375,549	19,375,333–19,375,196	138
Exon 5	19,372,135–19,372,837	19,372,644–19,372,465	180
Exon 6	19,368,938–19,369,503	19,369,197–19,369,340	144

**Table 2:** Primers selected after optimization for amplification of exons 2, 3, 5, and 6 in the koala CARD9 gene.

Exon	Primer	Sequence	Amplicon Size ^1^
Exon 2	Forward	5′-AGCTGATCTGCGCCAATGAC-3′	349 bp
	Reverse	5′-CATACCTGCCCACAGGTGCTT-3′	
Exon 3	Forward	5′-GCTGAGCCAGCTCTCCTTACT-3′	318 bp
	Reverse	5′-CAGTTTCTGGTCCAAACCAAGC-3′	
Exon 5	Forward	5′-AGCTGACCTACCTTCATGGA-3′	201 bp
	Reverse	5′-CCTCTTGCAGATAGACCGAC-3′	
Exon 6	Forward	5′-AGTGGTCAGTGTCTGTGTCC-3′	291 bp
	Reverse	5′-CATCATTCAGGCAGGAAGGTGG-3′	

^1^ Amplicon size is the length of sequence produced by each primer combination.

**Table 3:** Comparison of the exons used for each species for inter-species comparison of CARD9 sequences. The selected exons are based on alignments with exons investigated in koalas.

Species	Common Name	Koala Exon 2	Koala Exon 3	Koala Exon 5	Koala Exon 6
*Trichosurus vulpecula*	Common Brushtail Possum	Exon 1	Exon 2	Exon 4	Exon 5
*Sarcophilus harrisii*	Tasmanian Devil	Exon 2	Exon 3	Exon 5	Exon 6
*Vombatus ursinus*	Common Wombat	Exon 4	Exon 5	Exon 7	Exon 8
*Monodelphis domestica*	Grey Short-Tailed Opossum	Exon 3	Exon 4	Exon 6	Exon 7
*Mus musculus*	Mouse	Exon 2	Exon 3	Exon 5	Exon 6
*Homo sapiens*	Human	Exon 2	Exon 3	Exon 5	Exon 6

**Table 4:** Distribution of epidemiological variables and status of *Cryptococcus* infection in 22 koalas included in this study.

Sample ID	Animal Location	State	Number of Times Tested for *Cryptococcus*	If *Cryptococcus* Status Changed, How?	*Cryptococcus* spp. from Nasal Cavity ^1^	*Cryptococcus* Interaction ^2^	Exons Analyzed/Sequenced (Exons 2, 3, 5, 6)	Wild/Captive
USYD095F	Gunnedah	NSW	5		N/A	Not detected	2, 3, 5, 6	Wild
USYD062F	Gunnedah	NSW	3		N/A	Not detected	2, 5, 6	Wild
USYD038F	Gunnedah	NSW	5		N/A	Not detected	2, 3, 5, 6	Wild
USYD091M	Gunnedah	NSW	5		N/A	Not detected	2, 3, 5, 6	Wild
USYD085F	Gunnedah	NSW	5		N/A	Not detected	2, 3, 5, 6	Wild
USYD084M	Gunnedah	NSW	1		N/A	Not detected	2, 3, 5, 6	Wild
USYD064M	Gunnedah	NSW	5		N/A	Not detected	2, 3, 5, 6	Wild
USYD043M	Gunnedah	NSW	2	Progressed	*C. neoformans*	Colonized	2, 3, 5, 6	Wild
USYD085M	Gunnedah	NSW	2		*C. gattii*	Colonized	2, 3, 5, 6	Wild
USYD082M	Gunnedah	NSW	2		*C. neoformans*	Colonized	2, 3, 5, 6	Wild
USYD006M	Gunnedah	NSW	5	Recovered	*C. gattii*	Colonized	2, 3, 5, 6	Wild
DECC015M	Gunnedah	NSW	1		*C. neoformans*	Colonized	2, 3, 5, 6	Wild
USYD075F	Gunnedah	NSW	5	Re-occurring	*C. gattii*	Colonized	2, 3, 5, 6	Wild
USYD057M	Gunnedah	NSW	1		*C. gattii*	Colonized	2, 3, 5,6	Wild
USYD093M	Gunnedah	NSW	5	Recovered	Unknown	Subclinical	2, 3, 5, 6	Wild
DECC198F	Gunnedah	NSW	2		*C. gattii*	Subclinical	2, 3, 5, 6	Wild
DECC104F	Gunnedah	NSW	1		Unknown	Subclinical	2, 3, 5, 6	Wild
USYD014M	Gunnedah	NSW	2		Unknown	Subclinical	2, 3, 5, 6	Wild
DECC137F	Gunnedah	NSW	1		*C. gattii*	Subclinical	2, 5, 6	Wild
DECC071M	Gunnedah	NSW	2	Progressed	*C. gattii*	Subclinical	3, 5, 6	Wild
14-1684	Port Stephens	NSW	1		Unknown	Clinical Disease	6	Wild
21-1137	Port Stephens	NSW	1		Unknown	Clinical Disease	5, 6	Wild

^1^ Nasal colonization by *Cryptococcus* was detected using fungal culture of nasal swabs; Colonizing *Cryptococcus* spp. were identified using matrix-assisted laser desorption/ionization-time of flight (MALDI-TOF) mass spectrometry (MS). ^2^ Nasal colonization by *Cryptococcus* was detected using fungal culture of nasal swabs; Subclinical cryptococcosis was detected using Lateral Flow Assay (LFA) on serum samples, with positive tests confirmed by latex cryptococcal antigen agglutination test (LCAT). Clinical cryptococcosis was detected by the presence of intralesional cryptococcal yeasts at necropsy.

**Table 5:** Number of homozygous koalas sequenced at each SNP loci presenting homozygous wild-type (H_0_ WT), heterozygous (H_1_), or homozygous alternative (H_0_ Alt).

SNP	H_0_ WT	H_1_	H_0_ Alt	SNP Present (n/N) *
NW_018343963.1:19,355,780G > A(p.S23S)	8	8	4	12/20
NW_018343963.1:19,369,090C > T(intron)	14	7	1	8/22
NW_018343963.1:19,369,149G > T(intron)	7	5		5/12
NW_018343963.1:19,369,150C > A(intron)	8	4		4/12
NW_018343963.1:19,369,152C > T(intron)	14	2		2/16
NW_018343963.1:19,369,197C > G(p.K317N)	9	11		11/20
NW_018343963.1:19,369,211C > T(p.A313T)	12	8		8/20
NW_018343963.1:19,369,212C > A(p.E312N)	9	11		11/20
NW_018343963.1:19,369,244A > T(p.F302I)	15	4		4/19
NW_018343963.1:19,369,260T > G(p.E296H)	11	5		5/16

* Number of koalas with an SNP present as either homozygous or heterozygous genotype in relation to all koalas sequenced at that SNP loci.

**Figure A1:**
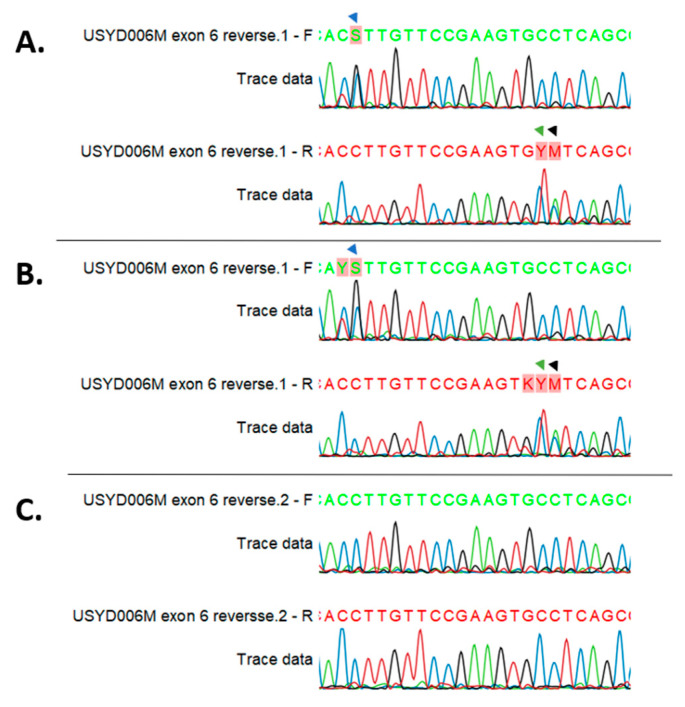
Excerpts from Sanger sequencing chromatograms of SNPs NW_018343963.1:19,369,197C > G(p.K317N) (Blue arrow), NW_018343963.1:19,369,211C > T(p.A313T) (Green arrow), and NW_018343963.1:19,369,212C > A(p.E312N) (Black arrow) in exon 6 of koala USYD006M when originally sequenced with Reverse.1 primer (**A**); re-sequenced using the Reverse.1 primer (**B**); and re-sequenced a Reverse.2 (**C**). Chromatograms show all SNPs were present when re-sequenced using the same Reverse.1 primer, but absent when the alternative Reverse.2 primer was used.

## Data Availability

The original contributions presented in the study are included in the article, further inquiries can be directed to the corresponding author.

## Appendix A

**Table A1:** Primers designed for each exon. Two forward and two reverse primers for exons 2, 3, 5 and 6 with sequence and length of primer.

Exon	Primer Direction	Sequence 5′-3′	Primer Length (nt)
2	Forward 1	AGCTGATCTGCGCCAATGAC	20
	Forward 2	AGGCATGGTTAGAGAAGGAGG	21
	Reverse 1	CATACCTGCCCACAGGTGCTT	21
	Reverse 2	CTGCCCACAGGTGCTTTGTC	21
3	Forward 1	GCTGAGCCAGCTCTCCTTACT	21
	Forward 2	GCCAGCTCTCCTTACTCTCTGA	22
	Reverse 1	CAGCTCTGTCCAAGGGGAACA	21
	Reverse 2	CAGTTTCTGGTCCAAACCAAGC	22
5	Forward 1	AGCTGACCTACCTTCATGGA	20
	Forward 2	ACCTTCATGGAGCTCTCCAGTTC	23
	Reverse 1	CCTCTTGCAGATAGACCGAC	20
	Reverse 2	GCAGATAGACCGACTAAAGCAC	22
6	Forward 1	AGTGGTCAGTGTCTGTGTCC	20
	Forward 2	TTGTTCCGAAGTGCCTCAGC	20
	Reverse 1	CATCATTCAGGCAGGAAGGTGG	22
	Reverse 2	GGCAGGAAGGTGGGACAAGA	20

## Appendix B

To determine the validity of these putative SNPs, three koalas had exon 6 re-sequenced twice, once with Reverse.2 primer as an alternative to the original primer pairing and then again with Reverse.1 primer (Table A1). All SNPs were absent when the Reverse.2 primer was used, yet all putative SNPs in each sample were reproducible and occurred in the same sequencing strands observed in previous sequencing reactions when re-sequenced with Reverse.1 primer (Figure A1). An Ensembl BLAST of exon 6 amplicon showed no other matches with more than 28bp other than the CARD9 gene in the koala genome.

**Table A2:** Primers used for validating putative SNPs in exon 6.

Exon	Primer	Sequence	Amplicon Size ^1^
Exon 6	Forward	5′-AGTGGTCAGTGTCTGTGTCC-3′	291 bp
	* Reverse.1	5′-CATCATTCAGGCAGGAAGGTGG-3′	
	** Reverse.2	5′-GGCAGGAAGGTGGGACAAGA-3′	282 bp

^1^ Amplicon size is the length of sequence produced by each primer combination. * Exon 6 Reverse.1 primer was used to sequence exon 6 in all koalas. ** Exon 6 Reverse.2 primer was used during troubleshooting of putative SNPs in exon 6 of three koalas.
